# P384: The effect of corrosion inhibition of the novel pre-cleaning spray detergent on stainless steel in the presence of chloride ions and its enhanced detergency

**DOI:** 10.1186/2047-2994-2-S1-P384

**Published:** 2013-06-20

**Authors:** Y Oda, Y Takashima, Y Hirata

**Affiliations:** 1 Biochemical laboratory, Saraya CO., Ltd, Osaka, Japan

## Introduction

In the reprocessing of medical devices, prevention of soil from drying and fixation is very important. Recently, pre-cleaning spray detergents are being used heavily to prevent soil from drying before cleaning operations. On the other hand, it is said that if soiled medical devices are not reprocessed immediately and left drying, corrosion occurs due to the presence of chloride ions contained in blood. With this, the effect of corrosion inhibition of the pre-cleaning spray detergent on stainless steel in the presence of chloride ions and its enhanced detergency were investigated.

## Objectives

The effect of corrosion inhibition of the pre-cleaning spray detergent on stainless steel in the presence of chloride ions and its enhanced detergency were investigated.

## Methods

The effect of corrosion inhibition was observed in appearance of stainless steel test pieces (SUS420J2) after soaking in a pre-cleaning spray detergent solution containing sodium chloride for a week. Its effect on the detergency was then verified with test soils described in ISO/TS 15883-5. After application of test soils to the stainless steel test pieces, they were sprayed with pre-cleaning spray detergents followed by manual or automated (washer disinfector) cleaning.

## Results

Stainless steels corrode in the presence ofchloride ions, and depending on the type of pre-cleaning spray detergents, medical devices could corrode in the presence of chloride ions contained in blood. In the novel pre-cleaning spray detergent formulation, corrosion is still inhibited even if the amount of chloride ions are two times of that found in blood. Furthermore, as the corrosion is inhibited, detergency is also being enhanced.

## Conclusion

The formulation of the novel pre-cleaning spray detergent exhibited corrosion inhibition of stainless steel in the presence of chloride ions as the detergency is enhanced, making it an excellent choice for a pre-cleaning spray detergent.

## Disclosure of interest

None declared

